# Impact of Strategies for Mitigating Delays and Disruptions in Cancer Care Due to COVID-19: Systematic Review

**DOI:** 10.1200/GO.20.00632

**Published:** 2021-03-03

**Authors:** Rafael Leite Pacheco, Ana Luiza Cabrera Martimbianco, Felipe Roitberg, Andre Ilbawi, Rachel Riera

**Affiliations:** ^1^ Centro Universitário São Camilo (CUSC), São Paulo, Brazil; ^2^ Centre of Health Technology Assessment, Hospital Sírio-Libanês, São Paulo, Brazil; ^3^ Oxford-Brazil EBM Alliance, Petropolis, Brazil; ^4^ Programa de Pós-graduação em Saúde e Meio Ambiente, Universidade Metropolitana de Santos (UNIMES), Santos, Brazil; ^5^ Instituto do Câncer do Estado de São Paulo/HCFMUSP, World Health Organization (WHO), São Paulo, Brazil; ^6^ Department of Noncommunicable Diseases, World Health Organization (WHO) Headquarters, Geneva, Switzerland; ^7^ European Society for Medical Oncology (ESMO), Lugano, Switzerland; ^8^ Discipline of Evidence-Based Medicine, Escola Paulista de Medicina (EPM), Universidade Federal de São Paulo (UNIFESP), São Paulo, Brazil

## Abstract

**PURPOSE:**

Delays and disruptions in health systems because of the COVID-19 pandemic were identified by a previous systematic review from our group. For improving the knowledge about the pandemic consequences for cancer care, this article aims to identify the effects of mitigation strategies developed to reduce the impact of such delays and disruptions.

**METHODS:**

Systematic review with a comprehensive search including formal databases, cancer and COVID-19 data sources, gray literature, and manual search. We considered clinical trials, observational longitudinal studies, cross-sectional studies, before-and-after studies, case series, and case studies. The selection, data extraction, and methodological assessment were performed by two independent reviewers. The methodological quality of the included studies was assessed by specific tools. The mitigation strategies identified were described in detail and their effects were summarized narratively.

**RESULTS:**

Of 6,692 references reviewed, 28 were deemed eligible, and 9 studies with low to moderate methodological quality were included. Five multiple strategies and four single strategies were reported, and the possible effects of mitigating delays and disruptions in cancer care because of COVID-19 are inconsistent. The only comparative study reported a 48.7% reduction observed in the number of outpatient visits to the hospital accompanied by a small reduction in imaging and an improvement in radiation treatments after the implementation of a multiple organizational strategy.

**CONCLUSION:**

The findings emphasize the infrequency of measuring and reporting mitigation strategies that specifically address patients' outcomes and thus a scarcity of high-quality evidence to inform program development. This review reinforces the need of adopting standardized measurement methods to monitor the impact of the mitigation strategies proposed to reduce the effects of delays and disruptions in cancer health care because of COVID-19.

## BACKGROUND

With the evolution of the current COVID-19 pandemic, the need to maintain essential health services has been universally recognized by WHO and its member states during the Seventy-Third World Health Assembly, 2020.^1^ Identifying and implementing feasible strategies to mitigate delays, interruptions, or abandonment of cancer and other essential health services have become public health priorities alongside the response to the pandemic itself.

**CONTEXT**

**Key Objective**
What are the effects of mitigation strategies for delays and disruptions in cancer health care because of the COVID-19 pandemic?
**Knowledge Generated**
The effects of nine different mitigation strategies were identified and analyzed. Five comprised a set of multiple actions focused on change in cancer services, for which individual actions were not measured. Meanwhile, four strategies encompassed a single action directed to address patient- or system-related factors.
The only comparative analysis reported a 48.7% reduction observed in the number of outpatient visits to the hospital accompanied by a small reduction in imaging and an improvement in radiation treatments after the implementation of a multiple organizational strategy.
**Relevance**
The findings emphasize the infrequency of measuring and reporting mitigation strategies that specifically address patients' outcomes and thus a scarcity of high-quality evidence to inform program development.

There has been a strong consensus that essential cancer services should continue. In response, the oncologic professional societies, cancer nongovernmental organizations, and governments have therefore issued recommendations for adjustments to deliver cancer care under the safest and the most conscientious manner as possible.^2-6^ These recommendations encompass adaptive strategies focused on screening, diagnosis, treatment, and rehabilitation process aiming to reduce the disruptions and delays in cancer care.

Overall, a previous systematic review conducted by our group identified 38 different categories of delays and disruptions related to cancer care during the pandemic (Riera et al, manuscript submitted for publication). These barriers were described according to the structural driver as provider-, patient-, and/or context-related and include delays in diagnosis and treatment, interruptions or changes in planned treatment, health products stockouts, and reductions in personnel workload or availability. The multiplicity and diversity of these types of delays and disruptions highlight the importance of a multisectoral, multidimensional response and the importance of monitoring the impact of implemented strategies.

Herein, we conducted a systematic review to identify the available strategies for and their reported effects of mitigating delays and disruptions in cancer care because of the COVID-19 pandemic.

The purpose of this study was to identify the effects of mitigation strategies for delays and disruptions in cancer health care because of the COVID-19 pandemic.

The clinical question is, as structured through the PICO acronym, as follows:

 • (P, population): individuals with cancer or under investigation for cancer, oncology services, and system
 • (I, intervention): strategies for reducing the impact of delays and disruptions because of COVID-19 on cancer care
 • (C, comparator): no strategy and different strategies
 • (O, outcomes): patient-related outcomes (clinical, laboratory, and image) and economic or administrative outcomes related to cancer care

## METHODS

### Study Design and Setting

This systematic review was conducted by the Oxford-Brazil EBM Alliance in collaboration with the WHO. The study was conducted in accordance with the recommendations of the Cochrane Handbook for Systematic Reviews of Interventions.^7^ The protocol was prospectively registered at the PROSPERO database (register number CRD42020196872). The reporting was written following the PRISMA statement.^8^

### Criteria for Including Studies

#### Types of studies.

Taking into account the research question of interest and the low likelihood of ideal randomized studies to answer it, we considered the following study designs:

 • Experimental studies (randomized, quasi-randomized, and nonrandomized trials; single experimental cohort, or controlled before-and-after studies)
 • Observational longitudinal comparative studies (cohort or case-control)
 • Observational noncomparative studies (case series or case studies reporting the experience of a specific cancer service)
 • Cross-sectional studies (prevalence, survey, or analytical cross-sectional)
 • Uncontrolled before-and-after studies (including interrupted time series studies with two or more measures before and after the event of interest)

#### Types of participants and scenarios.

Adults or children with a confirmed diagnosis or under investigation for cancer were considered. Any type of oncology service, from administrative setting to patient assistance facilities, including tertiary levels, was considered as an eligible scenario for assessing the impact of mitigation strategies.

#### Types of strategies.

Mitigation strategies were defined as those directly focused on managing eight categories of delays and/or disruptions in cancer care: (1) treatment interruption, (2) treatment delay, (3) treatment change, (4) reduction in the number of treatments, (5) diagnostic interruption, (6) diagnosis delay, (7) reduction in the number of diagnoses, and (8) healthcare service disruption (related to personnel, supplies, settings, etc).

On the basis of these, we considered the following strategies:

#### Treatment.

 • Radiotherapy—that is, change from conventional fractionation to hypofractionated scheme
 • Chemotherapy—that is, strategies to de-escalate, postpone, or change the regimens
 • Surgical procedures—that is, delay for low-grade or early-stage indolent neoplasms
 • Longer intervals for clinical reassessment

#### Diagnosis.

 • Cancer screening interval modification
 • Longer intervals for recurrence assessment

#### Healthcare services.

 • Creation of separate hubs for cancer care (COVID-free hubs)
 • Remote (telehealth or telephone appointments for new cases and follow-up)
 • Triage strategies for prioritizing procedures
 • Same-day procedures

#### Types of outcomes.

##### Primary outcomes.

 • Patient-related outcomes, including but not limited to overall survival, progression-free survival, response rate, toxicity, quality of life, or laboratory or image tests

##### Secondary outcomes.

 • Administrative or economic outcomes (including time from diagnosis to treatment start; frequency of interruptions in, abandonment of, or delays in current treatment; or volume of cancer-related visits, procedures, or hospitalizations)

We considered only studies addressing the effects of mitigation strategies for at least one outcome stated above. We assessed all outcomes reported at any time point. However, we would only pool similar time points together: short term (up to 1 month, inclusive) or long term (more than 1 month). When a study reports an outcome more than once in the same period, we would consider the last measurement.

### Criteria for Excluding Studies

We excluded any study or report that presented or proposed a mitigation strategy exclusively, with no measured impact regarding its consequence on the established primary or secondary outcomes. We did not consider studies addressing strategies focused exclusively on the prevention or treatment of COVID-19 among patients with cancer, such as the use of social distancing. Reports of a single individual case were not considered as well.

### Search for Studies

A comprehensive search of the literature was carried out using an electronic search with no restriction regarding the date, language, or status of the publication. Sensitive search strategies (Data Supplement) were developed for the following databases: CINAHL (Cumulative Index to Nursing and Allied Health Literature), Cochrane Library (via Wiley), EMBASE (via Elsevier), Epistemonikos,^9^ Health Systems Evidence,^10^ LILACS (Latin American and Caribbean Health Sciences Literature, via Biblioteca Virtual em Saúde), and MEDLINE (via PubMed).

An additional search was conducted in the following COVID-19–specialized sources: McMaster Daily News COVID-19,^11^ Oxford COVID-19 Evidence Service,^12^ and WHO—Global Literature on Coronavirus Disease.^13^

Additional nonstructured searches were conducted in the following cancer-specialized sources: ASCO Meeting Library (https://meetinglibrary.asco.org), ASCO Coronavirus Resources (https://www.asco.org/asco-coronavirus-information), ESMO COVID-19 and Cancer,^14^, and International Agency for Research on Cancer (IARC)—IARC research at the intersection of cancer and COVID-19.^15^

A search for gray literature was conducted in the OpenGrey database^16^ and medRxiv^17^ for preprint versions. A manual search was performed in the reference lists of the relevant studies.

### Selection of Studies

The selection process was conducted in a two-stage process supported by the Rayyan platform.^18^ In the first stage, two reviewers independently assessed all titles and abstracts retrieved by the search strategies. References identified as potentially eligible were then screened at the second stage, which involved the reading of the full text to confirm its eligibility. Any disagreement was solved by a third reviewer. Studies excluded in the second stage were presented in the excluded studies table along with the justifications for exclusions.

### Data Extraction

The procedure for data extraction was performed by two independent reviewers, and a preestablished data extraction form was adopted. Disagreements in this process were solved by a third reviewer.

### Methodological Quality of Studies

The methodological quality of the included studies was evaluated by two independent reviewers using validated tools for each study design as follows: (1) randomized controlled trial—Cochrane Risk of Bias Table;^7^ (2) nonrandomized trial, quasi-randomized trial, cohort study, or case-control study—ROBINS-I;^19^ (3) controlled before-and-after study—ROBINS-I with additional issues for (controlled) before-and-after studies;^19^ (4) uncontrolled before-and-after study (including interrupted time series)—ROBINS-I with additional issues for (uncontrolled) before-and-after studies;^19^ (5) analytical cross-sectional study—the Joanna Briggs Institute checklist for analytical cross-sectional studies^20^ (considering the eight questions to be answered, at the discretion of the review authors, the studies were categorized as presenting high quality (scored 7 or 8), moderate quality (scored 6 or 5), or low quality (scored 4 or lower); (6) prevalence cross-sectional study—the Joanna Briggs Institute checklist for prevalence studies;^21^ (7) case series—NIH Quality Assessment Tool for Case Series Studies;^22^ and (8) case study (service or system)—critical appraisal of qualitative studies of CEBM Oxford.^23^

### Unity of Analysis and Missing Data

The unit of analysis considered for this review was the same as assumed by the authors from primary studies included (generally aggregated as individual, group of individuals, healthcare system, or service). Considering the context requiring a rapid answer, the authors from primary studies were not contacted for missing data.

### Data Analysis and Presentation

Depending on data availability of clinical outcomes and homogeneity of studies, we planned to pool results from similar studies by random-effects meta-analyses (software Review Manager 5.4).

We planned to estimate the risk ratios (or odds ratios) and mean differences for dichotomous and continuous data, respectively. For time-to-event data, we planned to estimate the hazard ratio. A 95% CI was considered for the analyses.

Considering the scarcity and underreporting of data and the clinical and methodological heterogeneity among included studies, meta-analyses were not appropriated.

In this case, as previously defined, the results were presented as qualitative synthesis (descriptive presentation) through tables comprising the main findings of included studies and their methodological quality.

We planned to categorize the different strategies using the following parameters: (1) promoting agent (governmental organizations, nongovernmental organizations, private initiatives or policies, patient associations, research centers, or volunteers), (2) receiving agent (individual-oriented strategies, groups of individuals, healthcare services, or systems), (3) duration, and (4) delivery scheme (continuous or intermittent).

For making the report clearer and improving the applicability of the findings, different parameters were further added.

We considered data measured both after and before-and-after the adoption of any strategy during the COVID-19 pandemic. Therefore, to estimate the effect of a specific strategy, we considered both approaches: (1) data from noncomparative studies reporting pandemic data after strategy implementation and (2) data from comparative studies collating data obtained before and after the implementation of the strategy during the pandemic.

The comparison between prepandemic and pandemic data is the focus of a previous review developed by our work group (Riera et al, manuscript submitted for publication).

For noncomparative data, at our discretion, we categorized the findings according to their frequency: substantial delay or disruption when ≥ 50% of patients were affected, moderate when 10% to 49% of patients were affected, and low when ≤ 10% of patients were affected. For comparative data, we categorized the findings according to their reduction rate: substantial impact if the reduction rate of delays and disruptions was ≥ 50%, moderate impact if the reduction rate was from 10% to 49%, and low impact if the reduction rate was ≤ 10%.

### Heterogeneity Assessment

Methodological and clinical diversity of included studies would be considered to decide whether meta-analyses should be conducted or not. The existence of statistical heterogeneity would be evaluated by the χ^2^ test and its extension by the *I*^2^ test (*I*^2^ ≥ 50% indicates high heterogeneity among studies).

### Additional Analyses

No additional analyses were performed since pooling studies in meta-analyses were not considered appropriate. Nevertheless, we present here what would have been done if the quantitative syntheses had been possible.

For primary outcomes, we planned to conduct subgroup analyses considering the following: (1) type of cancer (ie, head and neck, gynecologic, hematologic, etc) and (2) age of participants (children *v* adults). Additionally, we planned to conduct sensitivity analysis considering the following: (1) the exclusion of no peer-reviewed publications or reports, (2) fixed-effect versus random-effects model meta-analysis, (3) reporting of both results when fixed-effect meta-analysis led to a different result, and (4) the exclusion of studies at high risk of bias.

Investigation of publication bias assessment would be performed by visual inspection of funnel plots if more than 10 studies are included in a single meta-analysis.

### Certainty of the Final Body of Evidence

If comparative studies assessing clinical outcomes had been included, we would have assessed the certainty of evidence using the GRADE approach for patient-related outcomes and a summary of findings table would have been presented using the GRADEpro GDT platform. However, no study with these characteristics was identified.

## RESULTS

### Results From Search

**FIG 1:**
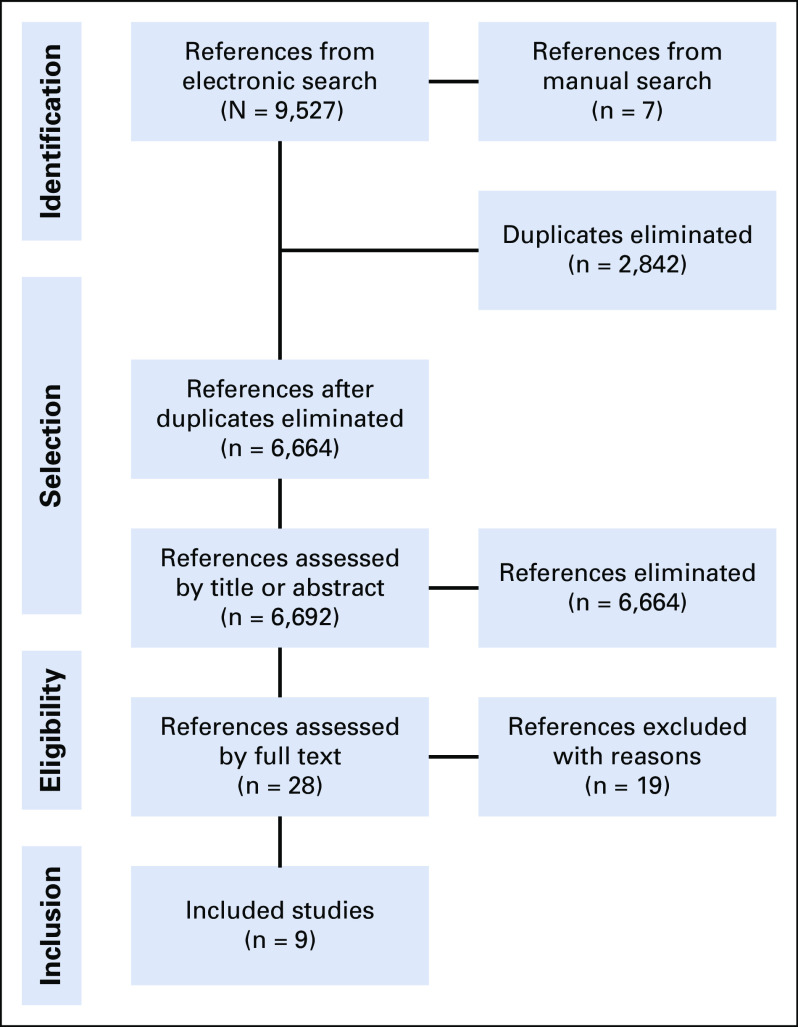
Flowchart of the process of study identification and selection.

We retrieved 9,527 references from the electronic search and seven additional references from the manual search. After excluding 2,842 duplicates, we screened the titles and abstracts of 6,692 references, excluded 6,664 that did not comprise the eligibility criteria, and selected 28 for full-text assessment. We excluded 19 studies after full-text reading, and the reasons for exclusion are presented in the Data Supplement.^24-42^ Nine studies fully met our inclusion criteria.^43-51^ The flowchart of the process of study identification and selection is presented in Figure 1.

### Characteristics of Included Studies

We identified two case series,^49,51^ three cross-sectional studies,^45-47^ and four analytical cross-sectional studies.^43,44,48,50^

The comparison groups referred to data before versus during the pandemic in three of the four analytical cross-sectional studies where the impact of the pandemic on health care was assessed but not the impact of any mitigation strategy. Thus, we considered only data obtained during the pandemic, and therefore such studies were analyzed as cross-sectional (not analytical).^43,44,50^

One of nine studies used a comparison group to estimate the effect of exposure to a specific strategy.^50^

The mitigation strategies were implemented in the United States, Italy, and China (two studies each) and Spain, the United Kingdom, and Iran (one study each). Sample sizes ranged from 15^51^ to 585.^50^ The strategies target health care for nonspecified types of cancers,^44,48-50^ breast cancer,^47-51^ head and neck cancer,^43,45^ and lung cancer.^46^

The Italian Ministry of Health provided funding for two studies, and for the remaining studies, no funding source was reported.^43,46^

Detailed information about the characteristics and findings of included studies is presented in Table 1.

**TABLE 1:**
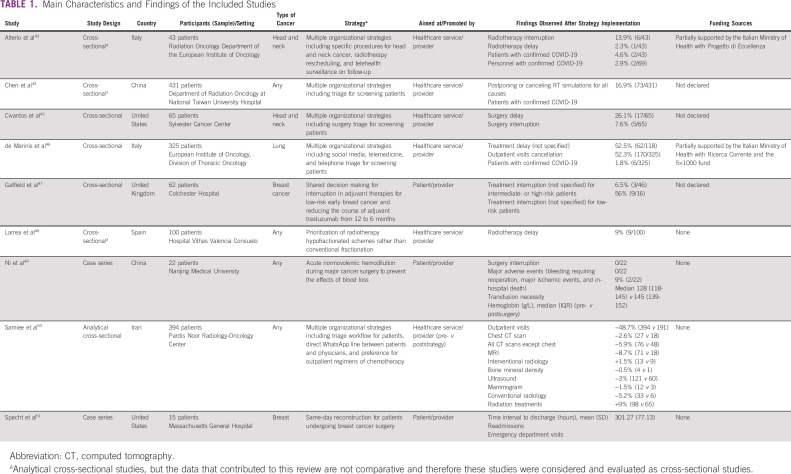
Main Characteristics and Findings of the Included Studies

### Results of Included Studies

Table 2 presents the description of strategies addressed by studies along with a categorization of the findings considering the possible impact of the strategies for mitigating delays and disruptions.

**TABLE 2:**
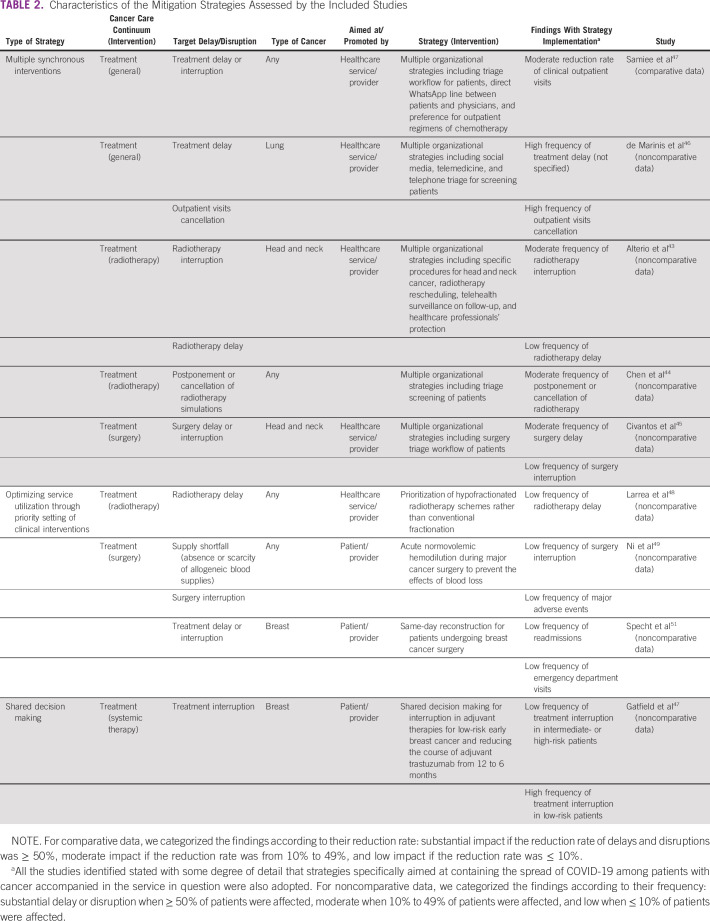
Characteristics of the Mitigation Strategies Assessed by the Included Studies

### Methodological Assessment of Studies

Methodological quality assessment of the included studies and reasons for judgment are presented in Table 3. The methodological quality was considered low^49^ to moderate^51^ for case series and low for all cross-sectional and analytical cross-sectional studies.

**TABLE 3:**
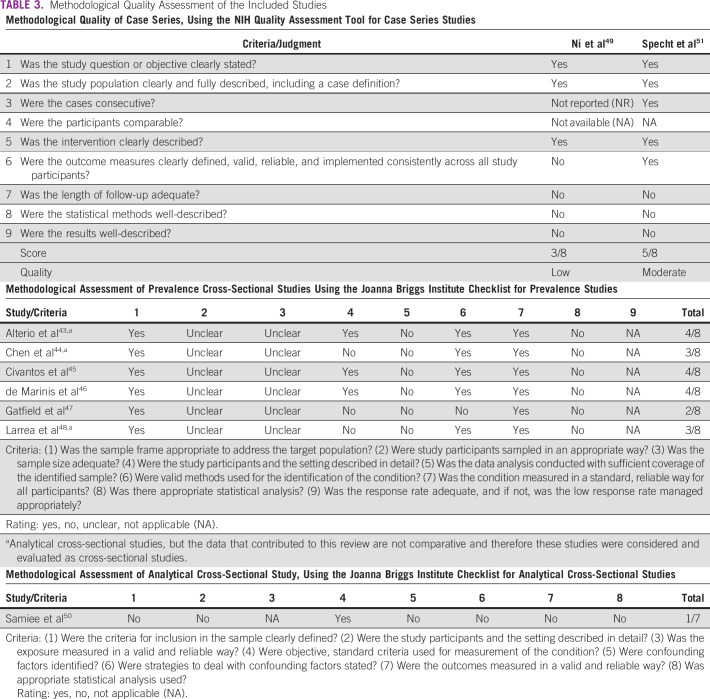
Methodological Quality Assessment of the Included Studies

## DISCUSSION

The primary finding of this systematic review is that despite the significant recent publications recommending how to maintain essential cancer services during the COVID-19 pandemic, mitigation strategies are frequently designed, developed, and described dissociated from expected outcomes of interest, and consensus best practices still rely on expert committees and the respective level of recommendation. As a result, only nine primary studies were available for this review. Of those, five comprised a set of multiple actions focused on change in cancer services, for which individual actions were not measured. Meanwhile, four strategies encompassed a single action directed to address patient- or system-related factors. It is important to note that specific measures to control the dissemination of COVID-19 among patients and personnel have been adopted in all studies.

In accordance with medical societies’ guidelines and recommendations, three studies reported mitigation strategies to establish tiered levels of urgency or priority for patients with cancer, tolerating delays in select low-risk patients both to prioritize resources for high-risk patients and to reduce the severity of population-level inferior outcomes. For example, cross-sectional studies reporting the adoption of multiple organizational strategies found an extremely varied type and frequency of treatment interruptions (eg, surgical care delays), not allowing one to predict the direction of a possible effect. Accordingly, assessing the degree of effectiveness of such strategies and distinguishing between planned or tolerated delays compared with unintended delays can be difficult, limiting the ability to assign value to studied interventions. Five of the nine studies adopted complex strategies, with several components or measurements taken. In these cases, it is not possible to isolate the effect of a single action for identifying the more effective one, neither replicating the strategies.

Four studies reported on the adoption of a single mitigation strategy generally focused on using a prioritization mechanism and/or optimizing service. For example, in one study, successful implementation of hypofractionated radiotherapy schemes found a low frequency of radiotherapy delay.^48^ Reducing the complexity of surgical services also had a positive impact on reducing subsequent healthcare service utilization. A study on same-day surgery reconstruction for patients with breast cancer revealed a low frequency of readmissions and emergency department visits.^51^ The use of acute normovolemic hemodilution during major cancer surgery to mitigate against the effects of blood supply scarcities resulted in a lower frequency of surgery interruptions and significant adverse events.^49^

The use of multiple organizational strategies focusing on routine communication with patients, maximizing remote support, and routine dialogue with patients was reported in five studies. Although cancellations were reported, these studies described a favorable benefit by significantly reducing disruptions to the continuity of cancer care services, such as radiotherapy or surgery cancellation.

Shared decision making has also been reported to maintain essential services, particularly for those at the highest risk for suffering an unfavorable outcome with treatment interruptions or delays. One study found that shared decision making in adjuvant therapies lowered the frequency of treatment interruption among intermediate- or high-risk patients.^47^ These studies have provided cross-sectional data that limited the ability to interpret, attribute, or suggest any generalizable negative or positive impact on overall cancer outcomes. A systematic approach to decision making and the resultant impact of health service study was reported by one study from Iran.^50^ Although there was a massive decrease in the outpatient visits (48.7%) and utilization of cancer-related imaging, the authors reported maintaining organized and consistent services according to patient needs during the pandemic.

This review has made clear that there is a lack of high-quality evidence in terms of meaningful clinical outcomes as a goal for the mitigation strategies adopted to address the major structural disruptions because of the COVID-19 pandemic, context-, patient-, or provider-related. The data synthesized mostly refer to surrogate end points. Although this was expected as studies that include outcome indicators will take a longer time to be completed, the majority of studies did not report the extent to which the reported end point of interest achieved expectations or value. Using predefined indicators, such as percentage of patients experiencing an unintentional delay or interruption in cancer services or reductions in supply stockouts or disruptions, will allow for more informative studies and generalizable conclusions.

To date, a small number of strategies found in this review focused on prevalent delays or disruptions reported in the literature. For example, our previous systematic review found that up to 75% of cancer care centers reported a lack of supplies during the pandemic (Riera et al, manuscript submitted for publication). However, in this review of mitigation strategies, only one study reported impacts on health product stockouts disruption.^49^

The pandemic has also exacerbated stress on patient engagement and maintaining integrated, patient-centered care, which are critical pillars of comprehensive cancer care.^52^ Patient involvement and the inclusion of patient-reported outcomes or experience measures have also been limited. In this review, all included strategies were implemented by the facilities or cancer programs and none by patient's groups. Only one involved the patient actively in the decision-making process.^47^

Ideally, the effects of any health intervention, including mitigation strategies of interest, should be addressed through an experimental comparative study, recognizing that the feasibility of randomized controlled trials may be difficult in this context. We recognize the challenges to perform a sound experimental study enrolling different centers to address one specific strategy, which could be external validity. Nonetheless, such an approach would reduce the bias around noncontrolled studies, such as those included in this review.

The high clinical and methodological diversity between included studies precluded any quantitative synthesis. Strategies probably affect the delays and disruptions differently in terms of specific cancer types and health systems. The study included a low number of countries with underdeveloped health systems and reduced surge capacity, highlighting the need to develop, implement, and measure mitigation strategies in those settings where the pandemic has had a more profound impact on service disruption.^53^

This systematic review has some limitations, including the risk of missing relevant studies that would potentially fulfill our inclusion criteria. Because of the fast-track publication of COVID-19 studies, there has been some incomplete indexing information on the databases, including lack of relevant keywords, missing abstracts, and change in DOI numbers. During the review's conduction, examples of dissonant information were found, including changes in the article title from the preprint version to the published version. All these aspects intensify the electronic search and selection process's complexity, increasing the risk of missing studies. Sensitive search strategies, additional searches in cancer and COVID-19 databases, and a double-checking selection process were carried out as attempts to minimize this risk.

Another limitation is that a subjective criterion was used to categorize the extension of the impact of mitigation strategies on delays and disruptions as high, moderate, and low. The criteria assumed were arbitrary and used by the author's discretion to make the findings more suitable for decision making. Different criteria may change the degree of strategy's impacts. Because of that, the magnitude of impact measurement should be interpreted as a broader perception of effect, and the results of the studies should be individually scrutinized.

This systematic review's findings should base larger and more appropriate studies for assessing the effects of the proposed strategies. The feasibility of different strategies should also be evaluated under other contexts as no included study was conducted in low- or lower- and middle-income countries.

To our knowledge, this is the first systematic review assessing the impact of strategies for mitigating delays and disruptions in cancer health care because of the pandemic. We hope our findings would contribute to foster further better-designed cancer research toward the interventions to reduce the burden of the pandemic over patients with cancer and healthcare systems.

In conclusion, this review identified nine strategies proposed for mitigating the outcomes related to the delays and disruptions in cancer health care because of COVID-19 and evaluated their impact for patients with cancer and health systems. Because of the limitation of methodological quality and inadequacy of the study designs, the effects of all the nine mitigation strategies addressed by the studies are not definitive. The findings emphasize the scarcity of high-quality and informative evidence to support the decision-making process appropriately and reinforce the need for better-designed studies aiming to assess the outcomes for the strategies proposed in the literature and directed for impactful delays and disruptions in cancer health care because of COVID-19.
